# Coupling Vibration Analysis of Trapped-Energy Rectangular Quartz Resonators by Variational Formulation of Mindlin’s Theory

**DOI:** 10.3390/s18040986

**Published:** 2018-03-26

**Authors:** Nian Li, Bin Wang, Zhenghua Qian

**Affiliations:** State Key Laboratory of Mechanics and Control of Mechanical Structures, College of Aerospace Engineering, Nanjing University of Aeronautics and Astronautics, Nanjing 210016, China; nianli_nuaa@163.com (N.L.); wangbin1982@nuaa.edu.cn (B.W.)

**Keywords:** rectangular plates, trapped-energy, quartz resonators, Mindlin’s theory, Ritz method

## Abstract

Mindlin’s two-dimensional theory has been derived and applied to research on quartz resonators for a long time. However, most works have focused on vibrations varying only in two directions, including thickness direction, while the effect of other directions like the length or width direction is normally neglected. Besides, researchers often model quartz resonators as fully electroded plates because of the resulting simplicity. Since a real device is finite in all directions and is only centrally electroded, results obtained in such works cannot offer quantitative information on vibrations with enough accuracy. In this paper, a theoretical analysis of a rectangular trapped-energy resonator of AT-cut quartz is studied using the Ritz method, associated with the variational formulation of Mindlin’s first-order equations. Frequency spectra and mode shapes of a real-scaled trapped-energy resonator, which is finite in all directions, are obtained with the consideration of mode couplings among thickness-shear mode, thickness-twist mode, and flexural mode. Results show the existence of an energy-trapping and coupling phenomenon and are helpful for thorough and accurate understanding of quartz resonator vibrations. Detailed discussions on the effects of structural parameters on mode couplings and energy trapping are provided, and the results can helpfully guide the selection of aspect ratio, length/thickness ratio, and electrode inertia in device design.

## 1. Introduction

Acoustic wave resonators made from piezoelectric crystals have been widely used in telecommunication and sensing. As frequency standards, resonators are often integrated in electrical circuits for time keeping, frequency operation, and signal processing. Moreover, since the resonant frequencies of resonators may change with various conditions like temperature or stress, resonators are made into acoustic wave sensors and are used for structural health monitors (SHMs), or quartz crystal microbalances (QCMs), etc. [1,2]. Various types of acoustic wave resonators can be found on the market. Based on the vibration modes, most resonators can be divided into two types. One is based on bulk acoustic waves (BAWs), and the other is based on surface acoustic waves (SAWs) [3]. Both of these types have broad applications. SAW resonators are commonly used in low frequency ranges due to their mature manufacturing techniques and low price. For the applications in the high frequency range, BAW resonators, which are smaller in size and have higher working frequencies, are used extensively. Though some new types of BAW resonators have come out, quartz is still the most widely used piezoelectric material for resonators because its advantages in certain aspects. A large number of quartz resonators operate with the so-called thickness-shear (TS) mode [4,5], which has been studied by many researchers and will be investigated in this paper as well.

Due to the anisotropy of quartz, theoretical analysis of crystal resonators using the typical three-dimensional theory of linear piezoelectricity presents considerable challenges. At present, theoretical results which are useful in resonator design can only be obtained for a few special cases, which need to make various approximations. One successful approach is to develop approximate, two-dimensional plate equations to simplify the problems so that theoretical analyses are possible. Two types of plate equations have been derived for resonator analysis, by Mindlin and Tiersten, respectively. Mindlin derived a system of two-dimensional equations for a fundamental TS mode with coupling to thickness-twist (TT) and flexural (F) modes [6,7]. These equations have been employed in the vibration analyses of quartz resonators for some time, and the results can indeed reveal some basic characteristics of the mode coupling and energy-trapping phenomenon [8,9,10,11,12]. However, most of these works only considered models geometrically varying in two directions like thickness and width or thickness and length. That is because difficulty and complexity still exist in the analyses for real-scaled resonators, even when using Mindlin’s two-dimensional equations. Therefore, only qualitative results were obtained for a basic understanding of mode couplings in quartz resonators. In [13], Wang and Yong developed a finite element method (FEM) based on Mindlin’s theory and analyzed the vibration of a rectangular quartz plate which is finite in three directions, but they did not extend their work to a partially electroded plate which is important for the analysis of the energy-trapping phenomenon. Theoretical analysis of partially electroded quartz plates is still challenging because of the emergence of continuity conditions between electroded and unelectroded regions, making the problem more complicated and hard to solve. Another two-dimensional approximate method was proposed by Stevens and Tiersten [14]. They derived two-dimensional scalar differential equations for the fundamental and the overtone TS modes only and neglected the influence of mode couplings. These equations show great accuracy for the pure TS mode and seem simple, which makes the method easy to utilize [15,16]. Shi, et al. analyzed trapped-energy quartz resonators with the Ritz method based on the variational formulation of Tiersten’s equation. Results in their paper show the existence of energy trapping for a rectangular plate, which is significant for the understanding of quartz resonator [17].

Based on the works using Mindlin’s theory and Tiersten’s equations, we can observe that both mode coupling and energy trapping are important in analyses of quartz resonators. However, there are currently no results taking into account mode coupling and energy trapping together for three-directional finite-size resonators. From [17], we know that the Ritz method is an effective global method for analyzing partial electroded rectangular resonators. Hence, in this paper we attempt to formulate Mindlin’s equation into a variational form and analyze coupling vibrations of trapped-energy resonators based on the Ritz method. By solving the eigenvalue problem described by the system of linear homogeneous equations, frequency spectra and mode shapes can be obtained and examined.

For free vibrations of quartz resonators, only traction-free conditions are needed, and hence there will be no more specific conditions for admissible functions. The general boundary conditions will be satisfied during the convergence process of the Ritz method. We choose Chebyshev polynomials as the admissible functions in our work because of their high accuracy, stable computation, and rapid convergence [18]. Based on literature research, we find that Ritz method is widely used in the vibration analysis of isotropic elastic plates [19,20,21,22], while for anisotropic plates like quartz, papers are rarely found. There are several works in which Ritz method based on Mindlin’s plate theory was used for the analysis of isotropic elastic plates, which can offer some guidance for the problem under question in this paper [21,23,24].

## 2. Mindlin’s First-Order Equations

**Figure 1:**
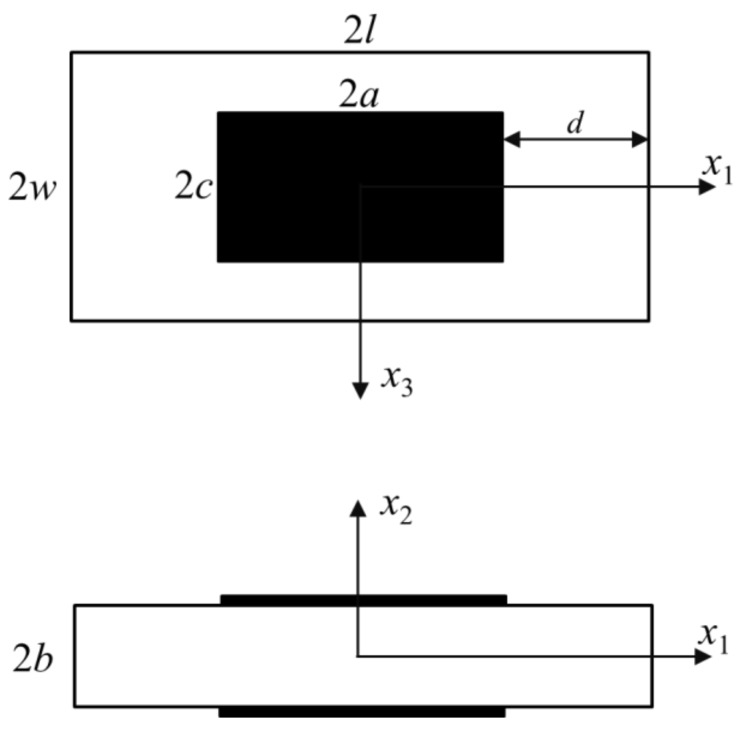
A rectangular partially electroded quartz resonator: plane view and cross section.

Consider a rectangular partially electroded quartz plate of thickness 2*b*, as shown in Figure 1. The *x*_2_ axis is determined from *x*_1_ and *x*_3_ by the right-hand rule. 2*l* and 2*w* are the length and width of the plate total length and width, respectively. For the electroded area, the length and width are defined as 2*a* and 2*c*. *d* in Figure 1 represents for the length of the outer unelectroded area.

There are different types of crystal cuts which have different performance characteristics. Specifically in this paper, we take the most commonly used AT-cut quartz for our research. The elastic constant matrix of AT-cut quartz is as follows [25]:

(1) $$C=\left[\begin{array}{cccccc} 86.74 & -8.25 & 27.15 & -3.66 & 0 & 0\\ -8.25 & 126.77 & -7.42 & 5.70 & 0 & 0\\ 27.15 & -7.42 & 102.83 & 9.92 & 0 & 0\\ -3.66 & 5.70 & 9.92 & 38.61 & 0 & 0\\ 0 & 0 & 0 & 0 & 68.51 & 2.53\\ 0 & 0 & 0 & 0 & 2.53 & 29.01 \end{array}\right]\times 10^{9\;}N/m^{2}$$

By neglecting the influence of the relatively small terms *c*_14_, *c*_24_, *c*_34_, and *c*_56_, vibration analysis of AT-cut quartz can be greatly simplified [6,26]. Mindlin’s first-order equations, which govern coupling modes between the TS mode, TT mode, and F mode, have been derived in [6]. For convenient use later, we briefly summarize the equations below. The equations for electroded and unelectroded regions are separated with a slight difference and we only need to give equations for the electroded region here. The displacement field is approximated by

(2) $$u_{2}=u_{2}^{\left(0\right)},\;u_{1}=x_{2}u_{1}^{\left(1\right)},u_{3}=x_{2}u_{3}^{\left(1\right)},$$

where $u_{2}^{\left(0\right)}$ is the F mode, $u_{1}^{\left(1\right)}$ is the TS mode, and $u_{3}^{\left(1\right)}$ is the TT mode. The relevant plate strains corresponding to Equation (1) are

(3) $$S_{4}^{\left(0\right)}=u_{2,3}^{\left(0\right)}+u_{3}^{\left(1\right)},\;S_{6}^{\left(0\right)}=u_{2,1}^{\left(0\right)}+u_{1}^{\left(1\right)},\;S_{1}^{\left(1\right)}=u_{1,1}^{\left(1\right)},\;S_{3}^{\left(1\right)}=u_{3,3}^{\left(1\right)},\;S_{5}^{\left(1\right)}=u_{3,1}^{\left(1\right)}.$$

The relevant stress components are

(4) $$\begin{array}{l} T_{4}^{\left(0\right)}=2bc_{44}S_{4}^{\left(0\right)},\\ T_{6}^{\left(0\right)}=2bc_{66}S_{6}^{\left(0\right)},\\ T_{1}^{\left(1\right)}=\frac{2b^{3}}{3}\left({\bar{c}}_{11}S_{1}^{\left(1\right)}+{\bar{c}}_{13}S_{3}^{\left(1\right)}\right),\\ T_{3}^{\left(1\right)}=\frac{2b^{3}}{3}\left({\bar{c}}_{31}S_{1}^{\left(1\right)}+{\bar{c}}_{33}S_{3}^{\left(1\right)}\right),\\ T_{5}^{\left(1\right)}=\frac{2b^{3}}{3}{\bar{c}}_{55}S_{5}^{\left(1\right)}, \end{array}$$

where ${\bar{c}}_{\alpha \beta }$ are determined by elastic constants with

(5) $${\bar{c}}_{\alpha \beta }=c_{\alpha \beta }-\frac{c_{\alpha 2}c_{2\beta }}{c_{22}},$$

and *α*, *β* can be taken from 1 to 6. The equations which govern three coupling modes are

(6) $$\begin{array}{l} T_{6,1}^{\left(0\right)}+T_{4,3}^{\left(0\right)}=\rho \left(1+R\right){\ddot{u}}_{2}^{\left(0\right)},\\ T_{1,1}^{\left(1\right)}+T_{5,3}^{\left(1\right)}-T_{6}^{\left(0\right)}=\frac{2b^{3}}{3}\rho_{1}\left(1+R\right){\ddot{u}}_{1}^{\left(1\right)},\\ T_{5,1}^{\left(1\right)}+T_{3,3}^{\left(1\right)}-T_{4}^{\left(0\right)}=\frac{2b^{3}}{3}\rho_{1}\left(1+R\right){\ddot{u}}_{3}^{\left(1\right)}, \end{array}$$

where *ρ* is the density of AT-cut quartz, *ρ*_1_ is used in Mindlin’s theory to correct the approximated two-dimensional equations and is determined by

(7) $$\rho_{1}=\frac{12\rho }{\pi^{2}},$$

*R* in Equation (6) represents the mass ratio of the electrode layers to the quartz layer and is defined by *R* = 2*ρ*^′^*b*^′^/*ρb*, where *ρ*^′^, 2*b*^′^ are the density and thickness of the electrode layer, respectively. Equations for the unelectroded region can easily be obtained by setting *R* to zero.

## 3. Variational Formulation

As preparation for the Ritz method, we need to rewrite Equation (6) into a variational form, which is

(8) $$\Pi =\underset{U+E}{\int }{\bar{\Pi }}_{u}dA-\underset{E}{\int }{\bar{\Pi }}_{u}dA+\underset{E}{\int }{\bar{\Pi }}_{e}dA,$$

where

(9) $$\begin{array}{l} {\bar{\Pi }}_{e}=bc_{44}\left(u_{2,3}^{\left(0\right)}+u_{3}^{\left(1\right)}\right)\left(u_{2,3}^{\left(0\right)}+u_{3}^{\left(1\right)}\right)+bc_{66}\left(u_{2,1}^{\left(0\right)}+u_{1}^{\left(1\right)}\right)\left(u_{2,1}^{\left(0\right)}+u_{1}^{\left(1\right)}\right)\\ +\frac{b^{3}}{3}\left({\bar{c}}_{11}u_{1,1}^{\left(1\right)}+{\bar{c}}_{13}u_{3,3}^{\left(1\right)}\right)u_{1,1}^{\left(1\right)}+\frac{b^{3}}{3}\left({\bar{c}}_{13}u_{1,1}^{\left(1\right)}+{\bar{c}}_{33}u_{3,3}^{\left(1\right)}\right)u_{3,3}^{\left(1\right)}+\frac{b^{3}}{3}{\bar{c}}_{55}\left(u_{3,1}^{\left(1\right)}+u_{1,3}^{\left(1\right)}\right)\left(u_{3,1}^{\left(1\right)}+u_{1,3}^{\left(1\right)}\right)\\ -\omega^{2}\left[\rho b\left(1+R\right)u_{2}^{\left(0\right)}u_{2}^{\left(0\right)}+\frac{b^{3}}{3}\rho_{1}\left(1+R\right)u_{1}^{\left(1\right)}u_{1}^{\left(1\right)}+\frac{b^{3}}{3}\rho_{1}\left(1+R\right)u_{3}^{\left(1\right)}u_{3}^{\left(1\right)}\right], \end{array}$$

and ${\bar{\Pi }}_{u}$ is almost the same as Equation (9) except *R* = 0. *U* and *E* in Equation (8) represent different integral regions. A simple treatment is employed in Equation (8) for convenience of calculation, which makes only three rectangular regions integrated and largely reduces the computing complexity. In our work, the integrals are calculated by the Gaussian quadrature method.

Equation (8) represents the total potential energy of the system. According to the minimum total potential energy principle, we have

(10) δΠ=0.

With successful substitution and derivation, the governing equations in Equation (6) can be obtained corresponding to the traction-free condition on the external boundary of unelectroded region as well as the continuity condition between the electroded and unelectroded regions. The validity of the variational formulation can be verified through this process.

## 4. Ritz Method

In this paper, we use the Ritz method based on the variational formulation of Mindlin’s equations to analyze the free vibration eigenvalue problem of the quartz resonator. We choose Chebyshev polynomial series as the admissible functions here due to their more rapid convergence and better stability. Besides, the Chebyshev polynomial and its derivatives can be expressed in simple and uniform form, which will reduce the coding effort [18]. As we know, the operating TS mode is symmetrical to both *x*_1_ and *x*_3_; other spurious modes possess different symmetries which can be determined from governing equations and are discussed by other researchers in some earlier works. The displacement functions are written in the form of duplicate series of Chebyshev polynomials, which are

(11) $$\begin{array}{l} u_{2}^{\left(0\right)}\left(\xi ,\eta \right)=\overset{\infty }{\underset{i=1}{\sum }}\overset{\infty }{\underset{i=1}{\sum }}A^{ij}P_{i}\left(\xi \right)Q_{j}\left(\eta \right)e^{i\omega t},\\ u_{1}^{\left(1\right)}\left(\xi ,\eta \right)=\overset{\infty }{\underset{m=1}{\sum }}\overset{\infty }{\underset{n=1}{\sum }}B^{mn}Q_{m}\left(\xi \right)Q_{n}\left(\eta \right)e^{i\omega t},\\ u_{3}^{\left(1\right)}\left(\xi ,\eta \right)=\overset{\infty }{\underset{p=1}{\sum }}\overset{\infty }{\underset{q=1}{\sum }}C^{pq}P_{p}\left(\xi \right)Q_{q}\left(\eta \right)e^{i\omega t}, \end{array}$$

where *ω* denotes the natural frequency of the quartz plate. *A^ij^*, *B^mn^*, and *C^pq^* are undetermined coefficients of each term. The *P* and *Q* standing for the antisymmetric and symmetric Chebyshev polynomial series, respectively, are

(12) $$\begin{array}{l} P_{s}\left(\chi \right)=\cos\left[\left(2s-1\right)\arccos\left(\chi \right)\right],\\ Q_{s}\left(\chi \right)=\cos\left[\left(2s-2\right)\arccos\left(\chi \right)\right],\;s=1,\;2,\;3,\ldots \end{array}$$

in which *χ* = *ξ*, *η*. *ξ* and *η* are the non-dimensional coordinates which are used here because both Chebyshev polynomials and Gaussian quadrature method are in the interval [−1, 1]. Relationships between the new non-dimensional coordinates and the original coordinates are

(13) $$\xi =\frac{x_{1}}{l},\;\eta =\frac{x_{3}}{w}$$

for the total rectangular plate, which is composed of the unelectroded region and the electroded region; or

(14) $$\xi =\frac{x_{1}}{a},\;\eta =\frac{x_{3}}{b}$$

for the inner rectangular plate which is for the electroded region only. Equations (13) and (14) are chosen separately based on the present integral region in Equation (8).

According to the minimum potential energy principle, we substitute Equation (11) into Equation (8) and can obtain the stationary conditions by minimizing the functional Π with respect to the coefficients of the admissible functions, i.e.,

(15) $$\frac{\partial \Pi }{\partial A^{ij}}=0;\;\frac{\partial \Pi }{\partial B^{mn}}=0;\;\frac{\partial \Pi }{\partial C^{pq}}=0.$$

Equation (15) leads to the following governing eigenvalue equation in matrix form

(16) $$\left(K-\Omega^{2}M\right)\left[\begin{array}{c} \left\{A\right\}\\ \left\{B\right\}\\ \left\{C\right\} \end{array}\right]=0,$$

where Ω represents the normalized resonant frequency of the trapped-energy quartz resonator and is defined as

(17) $$\Omega^{2}=\omega^{2}\left(\frac{2b}{\pi }\right)\frac{\rho }{c_{66}},$$

and Ω = 1 is the fundamental TS frequency of an infinite unelectroded plate with thickness 2*b*. The components of **K** and **M** are determined from Equation (15), which are not listed here.

## 5. Numerical Results and Discussion

As numerical examples, we consider the quartz resonator as shown in Figure 1, whose elastic constants have been given in Equation (1). The density of AT-cut quartz is *ρ* = 2649 kg/m^3^, and the thickness of quartz is chosen as 2*b* = 2 mm. We fix *a* = *c* and *l* = *w* here which will be convenient for later analysis. The number of terms of admissible functions needs to meet the convergence requirement and will be introduced in the specific examples later. The number of calculation points needed in the Gaussian quadrature method is closely related to the terms of admissible functions to get accurate integral results in Equation (8).

**Figure 2:**
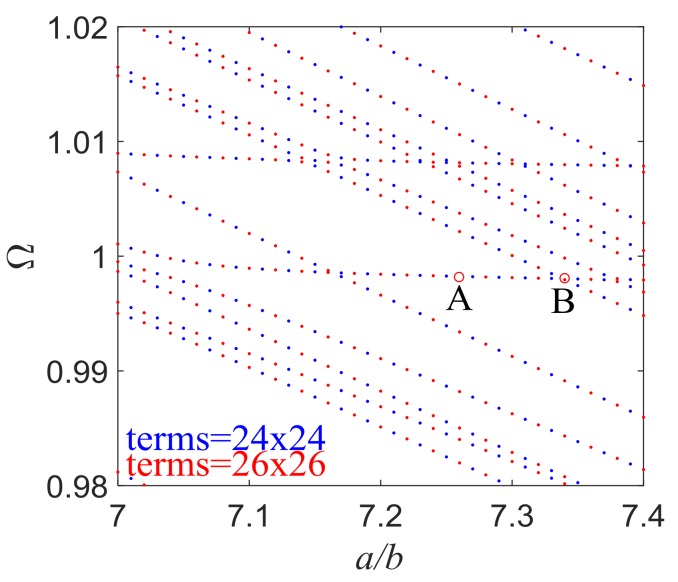
Frequency spectra of trapped-energy resonators when *d*/*b* = 15, *R* = 0.01.

Figure 2 shows the dimensionless frequency Ω versus the length/thickness ratio of the electroded region when *d*/*b* = 15 and *R* = 0.01. For the well-studied one-dimensional problems, similar figures are called frequency spectra and have important applications in mode coupling analysis. The frequency spectra are calculated with 24 and 26 terms of admissible functions in each direction, and both results are plotted in the figure with different color points to ensure the convergence and accuracy of current calculation. The same treatment is performed in latter analyses, where two series of points with different colors make up seemingly consecutive curves. Each data point in Figure 2 represents the frequency of a mode for a certain structure. Corresponding to a particular value of *a*/*b*, there are infinitely many modes. A few can be seen in the frequency range shown. It has been revealed in one-dimensional studies that the nearly flat parts of the curves near Ω = 1 in the frequency spectra represent the operating TS modes with weak couplings to other unwanted modes. When the flat parts begin to bend or seem to intersect with other curves, strong couplings begin to emerge, which is undesirable in device operation and should be avoided. Frequency spectra obtained in this paper are for the trapped-energy rectangular resonators, which can predict mode couplings more accurately.

**Figure 3:**
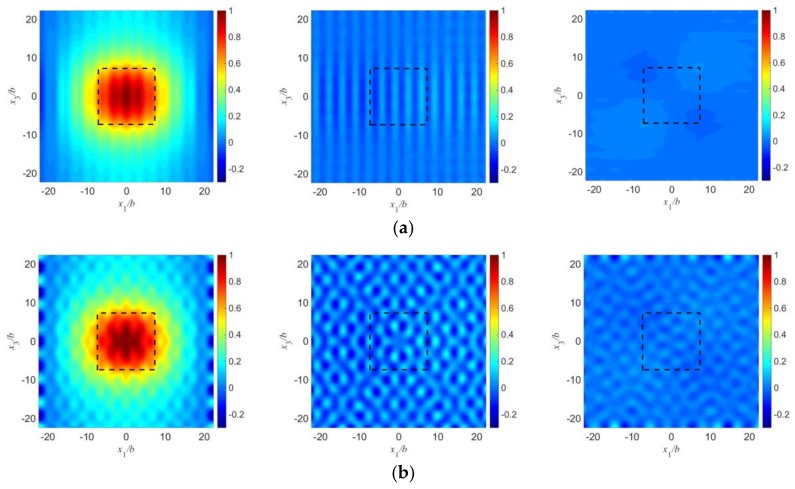
Essential thickness-shear (TS) modes with strong or weak mode coupling. (**a**) Point A: *a*/*b* = 7.26, *d*/*b* = 15, *R* = 0.01, Ω = 0.99824127; (**b**) Point B: *a*/*b* = 7.34, *d*/*b* = 15, *R* = 0.01, Ω = 0.99804365.

Figure 3 shows the mode shapes of $u_{1}^{\left(1\right)}$, $u_{2}^{\left(0\right)}$ and $u_{3}^{\left(1\right)}$ which represent the TS mode, F mode, and TT mode, respectively, corresponding to the two points labeled in Figure 2. Comparing Figure 3 with those mode shapes obtained in [17], we can find that due to mode coupling, mode shapes are not clean and smooth as shown in [17] since only TS modes were considered in their work. Mode shapes obtained here include an average long wave and a lot of short waves which are caused by spurious modes. Figure 3a is for point A which is in the middle of the flat part of the curves near Ω = 1. The three figures shown here are for the TS, F, and TT modes, respectively. Point A represents an essential TS mode. Ripples arising from short waves are unobvious in this case because coupling is weak at this point. For the TS mode, the vibration is mainly under the electroded region which is marked by the dashed line, and decays rapidly outside the electrodes. Near the plate edges there is little vibration left. The other two spurious modes do not show an energy-trapping phenomenon. Figure 3b is for point B which is at the end of a flat part. Still, energy trapping exists for the TS mode. Mode coupling is strong at this point which leads to obvious short waves compared with Figure 3a. The spurious modes become more obvious when coupling is strong, which will reduce the dominance of operating mode and decrease the working efficiency of quartz resonator. Hence, this mode is not as ideal as the mode in Figure 3a and needs to be avoided in design. Since we will focus our research on those essential TS modes and the amplitudes of displacement for F and TT modes are relatively small compared with TS mode (which can offer little useful information for us in later research), they will not be given later in this paper.

**Figure 4:**
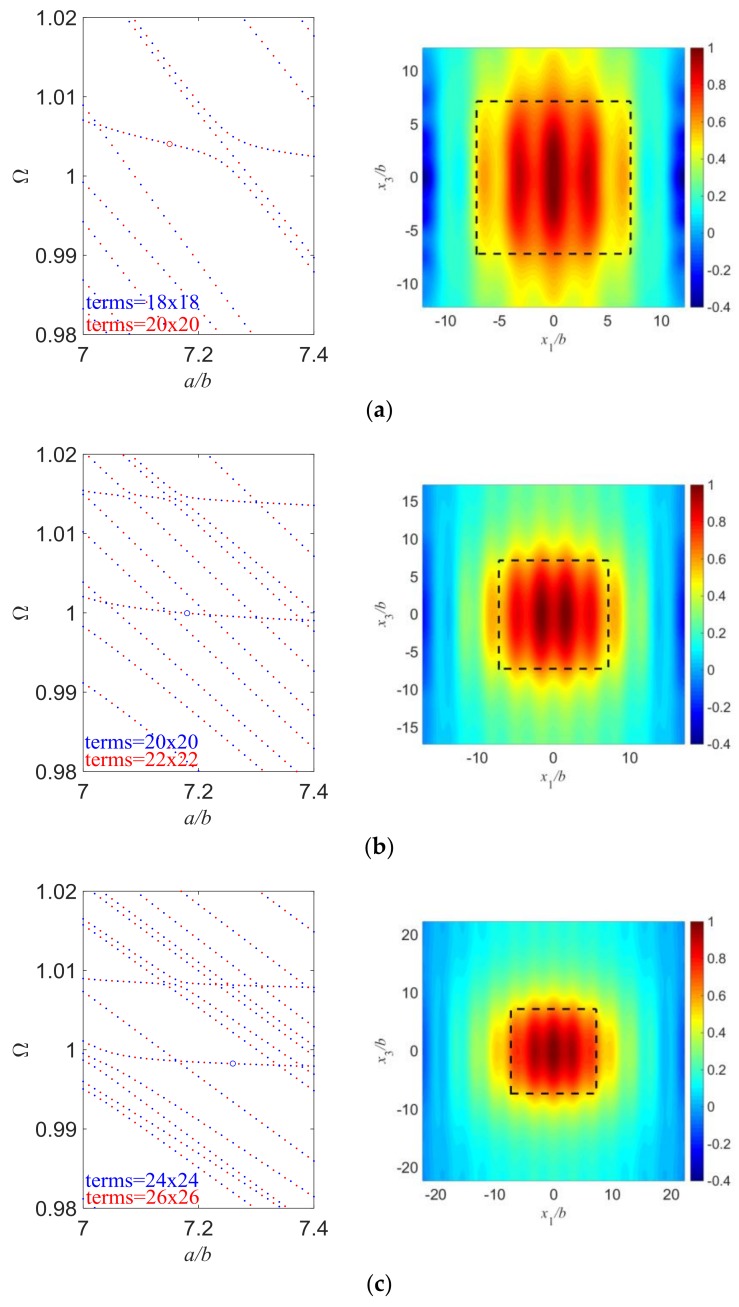
Essential TS modes with different *d*/*b*. (**a**) *a*/*b* = 7.15, *d*/*b* = 5, *R* = 0.01, Ω = 1.00404503; (**b**) *a*/*b* = 7.18, *d*/*b* = 10, *R* = 0.01, Ω = 0.99995715; (**c**) *a*/*b* = 7.26, *d*/*b* = 15, *R* = 0.01, Ω = 0.99824127.

In Figure 4, we change the value of *d*/*b* and try to keep everything else the same. Since mode coupling largely depends on the aspect ratio of the resonator, we need to study the frequency spectra first and choose those points with weak couplings so that we can get essential TS modes. Therefore, the length/thickness ratios of the electroded region have a slight difference in Figure 4, to exclude the influence of strong mode couplings. Also, the spectra are calculated with two different term numbers of admissible functions and plotted together to ensure the accuracy of the present results. For Figure 4a, the length of outer unelectroded region is not large enough and vibrations are still significant at the plate edge, which leads to great plate edge effects which need to be avoided. As *d*/*b* increases, vibrations near the plate edge become essentially zero, which means the edge effects are greatly decreased. We can conclude that to exclude the plate edge effects, a large enough plate needs to be used so that the vibration decays to essentially nothing before it reaches the plate edge. Besides, the resonant frequencies of essential TS modes slightly decrease with the increase of *d*/*b* which is as expected because the frequencies of the operating TS modes are mainly dependent on the plate thickness and weakly on the length, and larger plates have lower frequencies.

**Figure 5:**
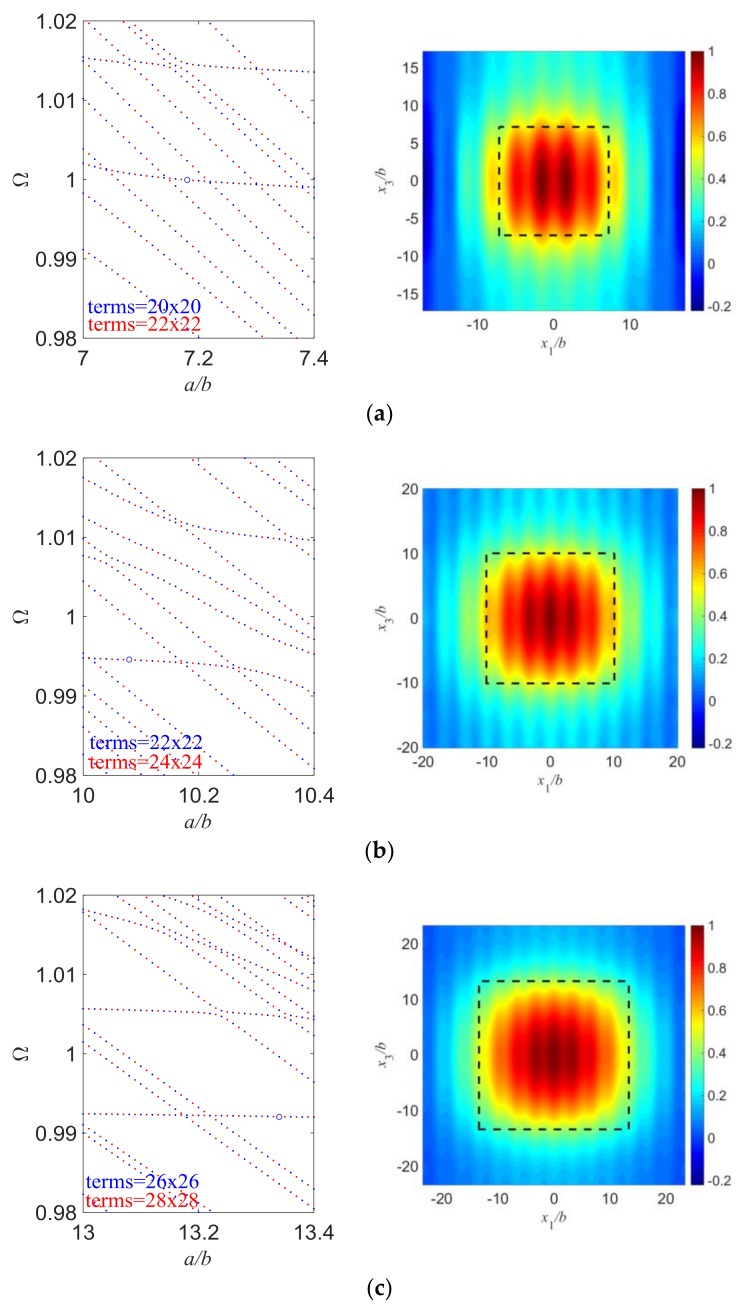
Essential TS modes with different *a*/*b*. (**a**) *a*/*b* = 7.18, *d*/*b* = 10, *R* = 0.01, Ω = 0.99995715; (**b**) *a*/*b* = 10.08, *d*/*b* = 10, *R* = 0.01, Ω = 0.99455566; (**c**) *a*/*b* = 13.34, *d*/*b* = 10, *R* = 0.01, Ω = 0.99207397.

Figure 5 shows several mode shapes with varying length/thickness ratios of the electroded region, and everything else is kept the same. The length of outer unelectroded region is fixed here to confirm the vibrations can decay to essentially zero near the plate edge. Clearly we can see that the vibration distribution closely follows the electrode, and becomes wider when the electrode dimension increases, which is also shown in Figure 4. Besides, mode coupling becomes weaker and the vibration distribution is more confined under the electroded region as *a*/*b* increases. A nearly pure TS mode can be obtained if the electrode dimension is wide enough. The resonant frequencies of essential TS modes slightly decrease with the increase of *a*/*b*.

**Figure 6:**
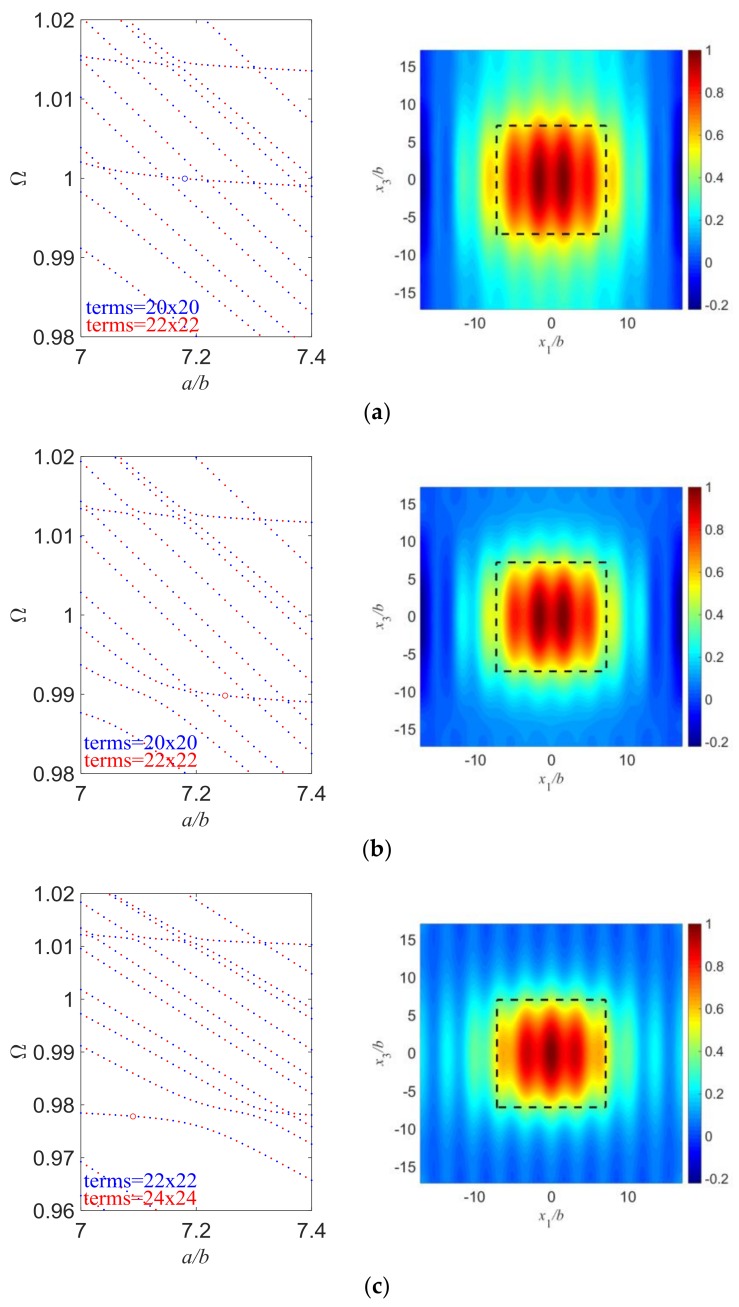
Essential TS modes with different *R*. (**a**) *a*/*b* = 7.18, *d*/*b* = 10, *R* = 0.01, Ω = 0.99995715; (**b**) *a*/*b* = 7.25, *d*/*b* = 10, *R* = 0.02, Ω = 0.98982614; (**c**) *a*/*b* = 7.09, *d*/*b* = 10, *R* = 0.03, Ω = 0.97784851.

Figure 6 shows the effect of the electrode inertia on the essential TS mode. All parameters are basically the same except that *R* is varied. Still, the values of *a*/*b* are slightly different because the aspect ratios of electroded region are chosen through frequency spectra to get essential TS modes. We can see in Figure 6 that more electrode inertia is associated with stronger energy trapping, as expected. Again, the frequency of the essential TS mode slightly decreases when electrode inertia increases.

**Figure 7:**
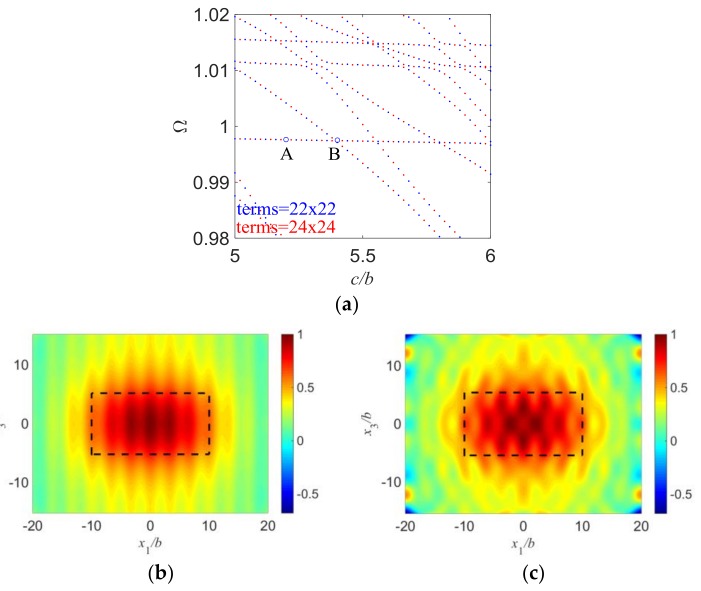
Influence of *c/b* on mode couplings. (**a**) Frequency spectra with different *c/b* when *a*/*b* = 10, *d*/*b* = 10, *R* = 0.01; (**b**) Point A: *c/b* = 5.20, *a*/*b* = 10, *d*/*b* = 10, *R* = 0.01, Ω = 0.99759004; (**c**) Point B: *c/b* = 5.40, *a*/*b* = 10, *d*/*b* = 10, *R* = 0.01, Ω = 0.99747716.

All the results shown above are about a square plate of *a* = *c* and *l* = *w* for the convenience of discussions. Since width effect is also an important factor that needs to be considered in the vibration analysis of quartz, some results for a rectangular plate with a varying *c/b* are given in Figure 7, where we set *a*/*b* = 10, *d*/*b* = 10, and *R* = 0.01 and study the width effects. Figure 7a is frequency spectra of quartz resonators with varying *c*/*b*. It looks similar to the frequency spectra discussed above, with flat parts near the fundamental TS frequency and similar characteristics. Figure 7b,c show mode shapes for two different vibration modes with weak and strong couplings, respectively. Like the results in Figure 3, those modes of the nearly flat parts in this frequency spectra represent the essential TS modes with weak mode couplings, while the modes of those end points of the flat parts represent vibration modes with strong mode couplings and need to be avoided in design. Those results can be clearly observed in Figure 7b,c.

## 6. Conclusions

A variational formulation for Mindlin’s first-order equations governing the TS mode coupled with TT and F modes is established in this paper. The Ritz method is applied for analyzing trapped-energy resonators based on the mentioned equations. Chebyshev polynomial series are employed here, which converge rapidly and can produce accurate frequencies with corresponding mode shapes. Numerical results show the existence of energy trapping and mode coupling. The structural parameters need to be carefully selected to avoid strong mode couplings. A large enough unelectroded area needs to be kept to ensure the vibration can decay essentially to zero. Resonators with longer electrodes show more ideal essential TS modes with weak couplings. Thicker electrodes are associated with more-trapped modes. Besides, the width/thickness ratio also has great influence on mode couplings and needs to be carefully chosen for quartz resonators. Those results obtained in this paper give the effects of both energy trapping and mode coupling in rectangular resonators, which are significant and helpful for device design.
